# Robust fat saturation applied to late enhancement

**DOI:** 10.1186/1532-429X-16-S1-P196

**Published:** 2014-01-16

**Authors:** Martin A Janich, Jeff A Stainsby, Glenn S Slavin, David Stanley, Christopher J Francois, Scott B Reeder, James F Glockner, Anja C Brau

**Affiliations:** 1 GE Global Research, Munich, Germany; 2 GE Healthcare, Toronto, Ontario, Canada; 3 GE Healthcare, Bethesda, Maryland, USA; 4 GE Healthcare, Rochester, Minnesota, USA; 5 Radiology, University of Wisconsin, Madison, Wisconsin, USA; 6 Mayo Clinic, Rochester, Minnesota, USA; 7 GE Healthcare, Munich, Germany

## Background

Late Gadolinium Enhancement (LGE) allows imaging of infarction and cardiomyopathies by measuring the accumulation of contrast agent within the myocardium. The shortened T_1 _relaxation time compared to healthy myocardium is imaged by an inversion recovery (IR) prepared segmented fast gradient echo sequence in which pathology and fat show as bright signal. The fat signal can lead to misinterpretation and poor visualization of epicardial enhancement. In previous work fat was suppressed by using two appropriately timed fat-selective RF pulses which re-invert and invert fat signal [Foo et al., JMRI, 2007] but this technique was sensitive to off-resonance and heart rate variations. The goal of the present work is to make two improvements to fat-saturated LGE: (1) increase robustness against B_0 _and B_1 _variations by using asymmetric adiabatic RF pulses, and (2) increase robustness against heart rate variations through dynamic timing of fat-selective RF pulses.

## Methods

**Figure 1:**
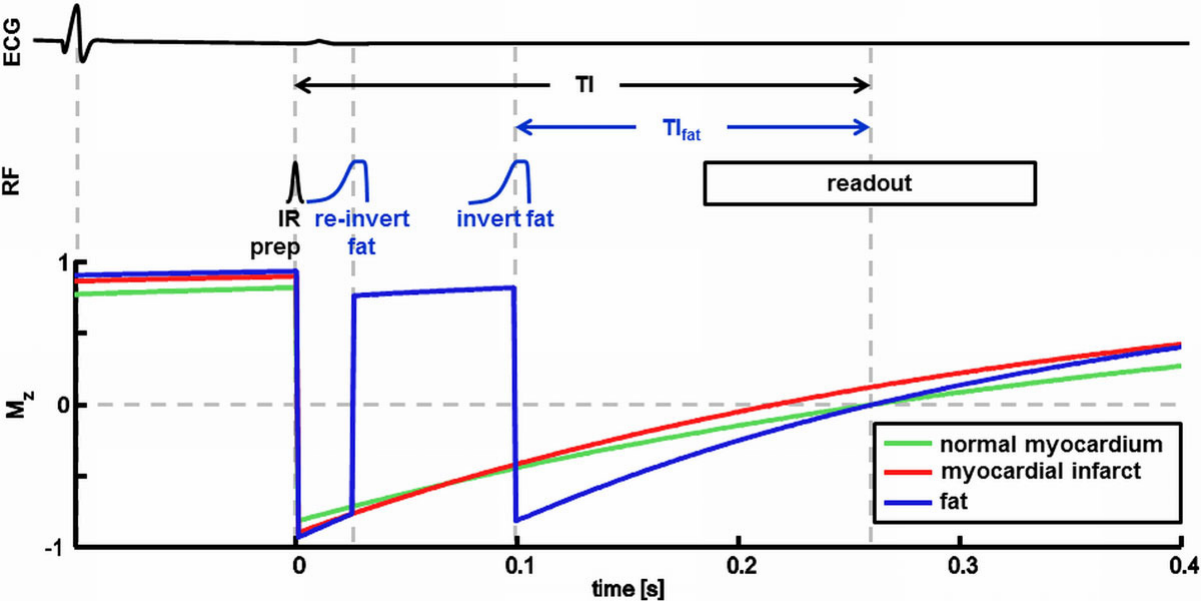
**Timing diagram of cardiac gated IR prepared fat saturation pulse sequence (RF phase not shown) and possible M_z _signal evolution**. Signal from normal myocardium is nulled by proper selection of TI and signal from fat is nulled by dynamic timing of TI_fat _which leads to heart rate independent fat suppression.

The selectivity of fat-selective pulses was improved by designing an asymmetric adiabatic inversion pulse based on HS1 and tanh/tan [Hwang et al., JMR, 1999]. This pulse had a transition width of 87 Hz which is 2 times narrower compared to a symmetric inversion pulse of the same maximum B_1 _(10μT) and pulse duration (40 ms). M_z _magnetization depends on the repetition time of the IR pulse and therefore in cardiac gated sequences depends on the patient heart rate. Heart rate variation leads to a shifting of the inversion time (TI_fat_) at which the fat signal is nulled. Therefore a heart rate independent nulling of fat signal was developed by measuring the length of each R-R interval during the examination and time-shifting the fat-selective inversion pulse accordingly (Figure 1). The method was tested in volunteers without contrast and patients with contrast on 1.5T (MR450w) and 3.0T (MR750) MR systems (GE Healthcare).

## Results

**Figure 2:**
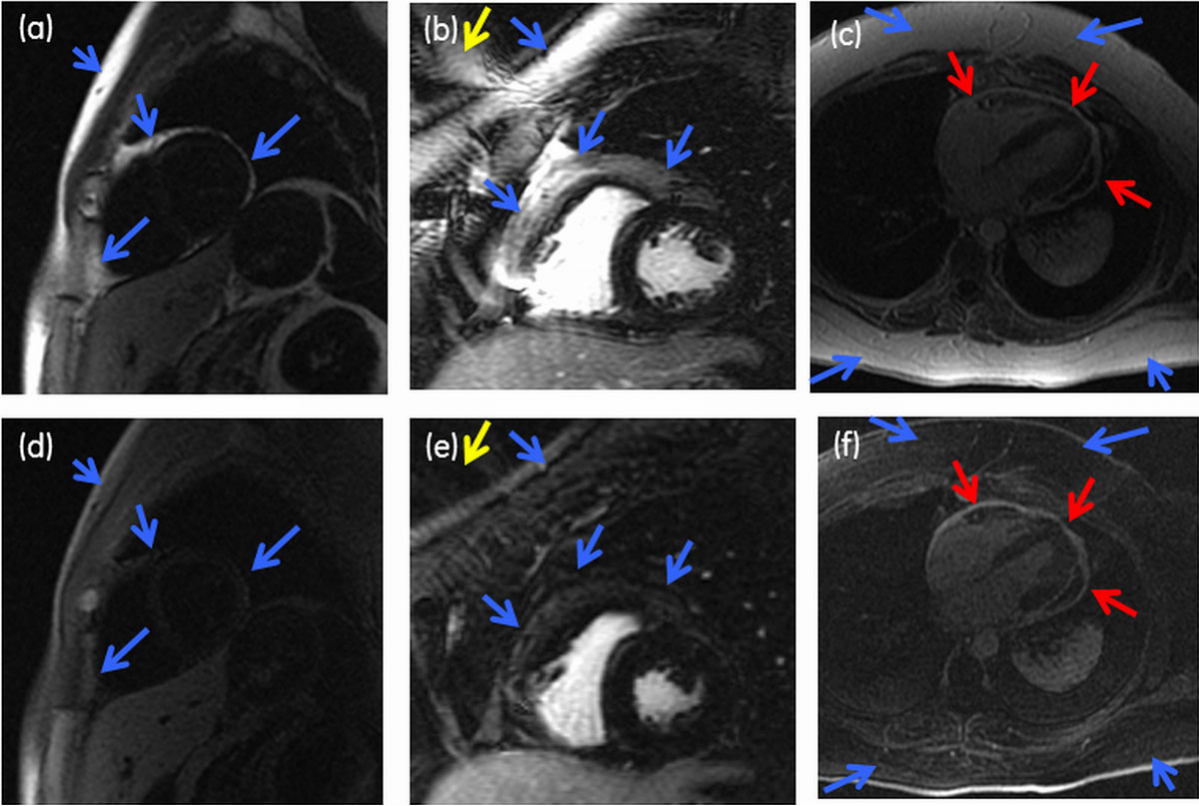
**Short-axis IR prepared images from the heart of a (a, d) healthy volunteer (w/o contrast, 1.5T, HR ≈60 bpm, TI 280 ms), (b, e) patient (w/contrast, 3.0T, HR ≈60 bpm, TI 290 ms), and (e, f) patient with pericardial enhancement (w/contrast, 1.5T, HR ≈80 bpm, TI 260 ms)**. The upper row images (a-c) were acquired with IR preparation only and the lower row images (d-f) were acquired with IR preparation and fat saturation. The new technique led to suppression of normal myocardium and fat signal (blue arrows) which avoided image wrapping of subcutaneous fat (e, yellow arrows) and better visualization of pericardial enhancement (f, red arrows).

The T_1,fat _value which influences the heart rate-dependent TI_fat _was calibrated in healthy volunteers at field strengths 1.5T (220 ms) and 3.0T (300 ms). With appropriate TI selection the technique led to nulling of signal from healthy myocardium and from fat. The cases without contrast injection had poor blood-myocardium contrast due to the lack of Gd (Figure 2(a)) but fat was successfully suppressed.

## Conclusions

Benefits of fat-saturated LGE imaging include avoiding image wrapping of subcutaneous fat and therefore reduced possible misdiagnosis (Figure 2(b)), and in patients with pericardial enhancement the ability to rule out epicardial fat and better visualization of pathology (Figure 2(c)). The selectivity improvement of fat-selective pulses was particularly important at 1.5T where the chemical shift difference between fat and water is only 220 Hz. The technique presented here was proven to be robust in more than 20 cases.

